# Supplementary material for: Proline Stickland fermentation supports C. difficile spore maturation
Source: Appl Environ Microbiol. 2025 Jun 4;91(7):e00551-25. doi: 10.1128/aem.00551-25 (PMC12285225; doi:10.1128/aem.00551-25)
Supplement: Supplemental figures — Figures S1 to S3. [file aem.00551-25-s0001.pdf]

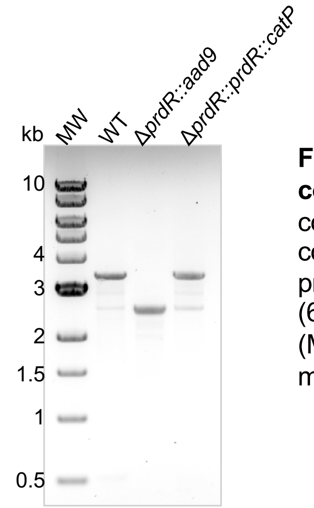

Figure S1. Mutant and complementation confirmation for *C. difficile prdR*. PCR confirmation of *prdR* deletion and complementation. Expected products using primers oMC2994/3311 are 3592 bp for WT (630Δ*erm*) and *prdR* complement (MC2668) and 2667 bp for the Δ*prdR* mutant (MC2337).

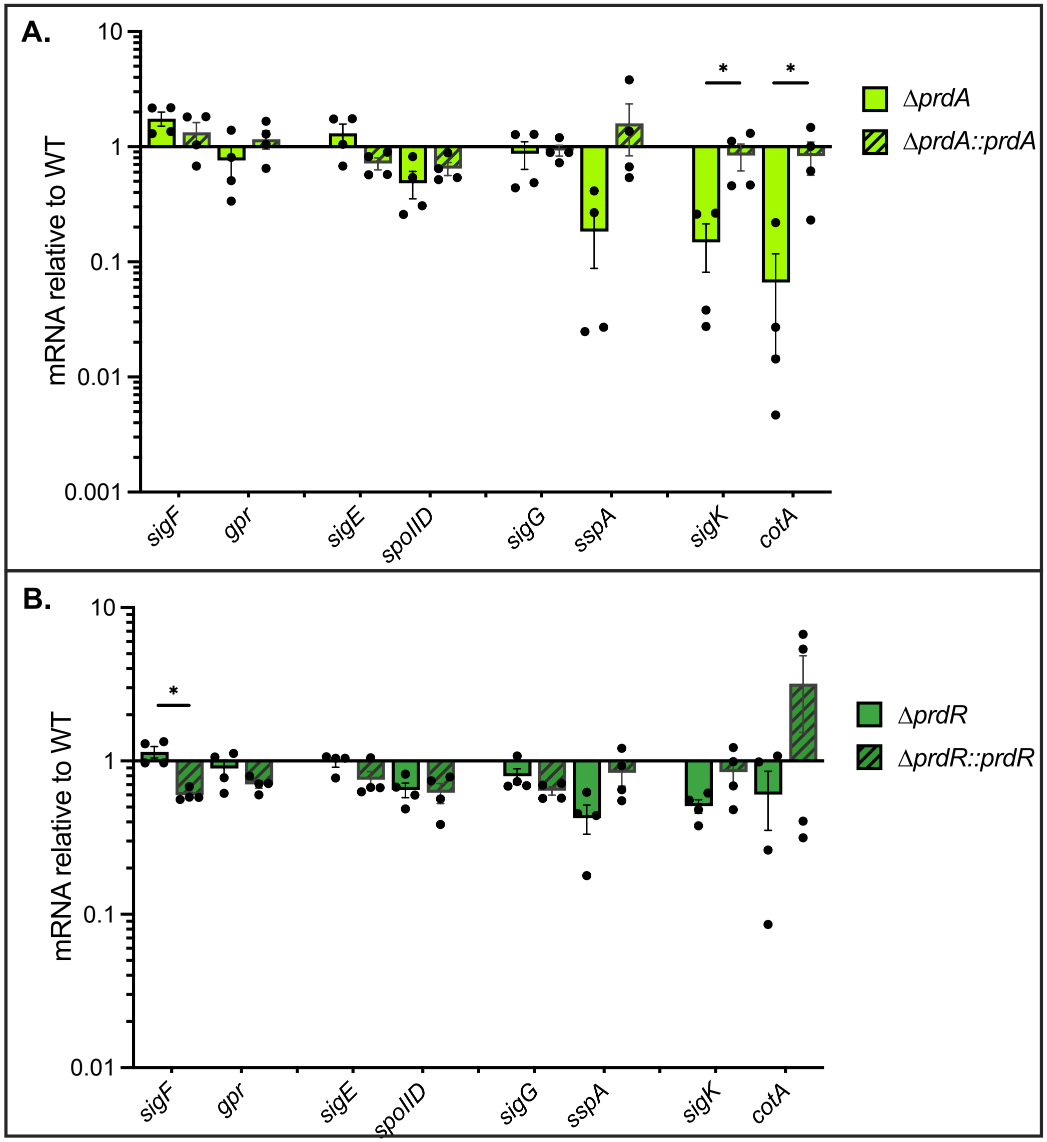

Figure S2. Complementation of *prdA* and *prdR* restores sporulation-specific gene expression. Quantitative reverse transcription PCR analysis of sporulation-specific genes temporally expressed during spore development for **A)** Δ*prdA* (MC2773) or the Δ*prdA::prdA* complement (MC2774) and **B)** Δ*prdR* (MC2337) or the Δ*prdR::prdR* complement (MC2668) grown on 70:10 sporulation agar for 12 h, relative to the parent strain. The means, individual values, and standard error of the mean are shown for four biological replicates. Data were analyzed by unpaired Student's *t*-test comparing the mutants and complemented strains. *P <0.05, **P <0.01

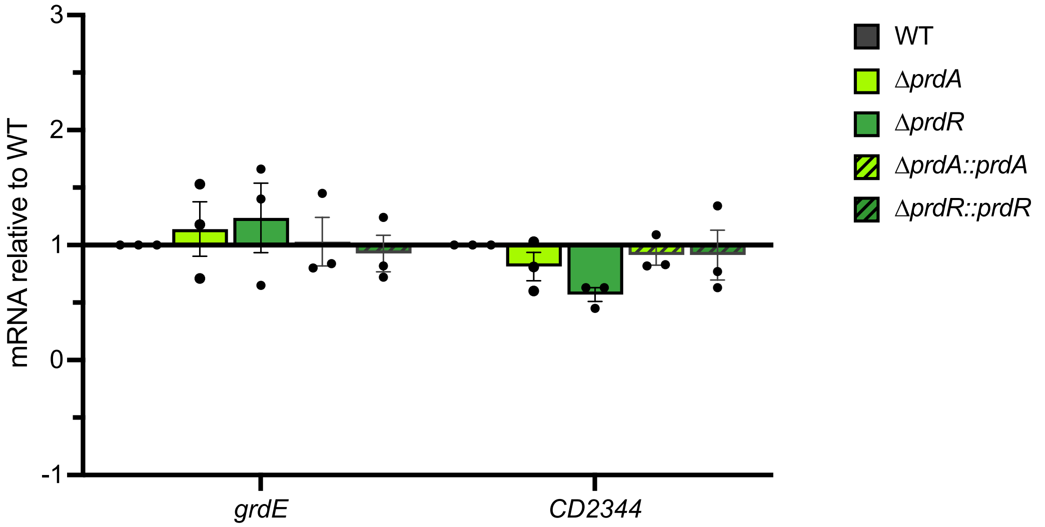

Figure S3. Rex-dependent (redox) gene expression is unaffected by deletion of *prdA* or *prdR*. Quantitative reverse transcription PCR analysis of redox regulated genes for wild-type (WT, 630Δ*erm*), Δ*prdA* (MC2773), the Δ*prdA::prdA* complement (MC2774), Δ*prdR* (MC2337), and the Δ*prdR::prdR* complement (MC2668) grown on 70:10 sporulation agar for 12 h, relative to the parent strain 630Δ*erm*. The means, individual values, and standard deviation for three biological replicates are shown. Data were analyzed by two-way ANOVA respective to the parent strain. No statistically significant differences were observed.
