# Supplementary material for: Proline Stickland fermentation supports C. difficile spore maturation
Source: Appl Environ Microbiol. 2025 Jun 4;91(7):e00551-25. doi: 10.1128/aem.00551-25 (PMC12285225; doi:10.1128/aem.00551-25)
Supplement: Table S1 — Vector construction. [file aem.00551-25-s0002.pdf]

Table S1. Vector Construction

Plasmid	Construction details
pMC1223	*prdR* deletion construct containing an 814 bp homology arm 5' of *prdR* (*CD3245*) and an 802 bp 3' homology arm were amplified with primers oMC2990/oMC2991 and oMC3097/3098, respectively. A 1051 bp *aad9* cassette from pRT1099 was amplified with primers oMC2943/2944, and all fragments were Gibson assembled into pMSR.
pMC1402	An allelic exchange *prdR* knock-in construct carrying a 2685 bp 5' arm with the *prdR* promoter and *prdR,* followed by a 769 bp *catP* cassette and a 753 bp 3' homology arm was cloned into pMSR-bla by Genscript (Piscataway, NJ).
